# Does wild-type Cu/Zn-superoxide dismutase have pathogenic roles in amyotrophic lateral sclerosis?

**DOI:** 10.1186/s40035-020-00209-y

**Published:** 2020-08-19

**Authors:** Yoshiaki Furukawa, Eiichi Tokuda

**Affiliations:** 1 Department of Chemistry, Laboratory for Mechanistic Chemistry of Biomolecules, Keio University, 3-14-1 Hiyoshi, Kohoku, Yokohama, Kanagawa 223-8522 Japan; 2 Present address: Laboratory of Clinical Medicine, School of Pharmacy, Nihon University, 7-7-1 Narashinodai, Funabashi, Chiba, 274-8555 Japan

**Keywords:** Amyotrophic lateral sclerosis, Cu/Zn-superoxide dismutase, Protein misfolding

## Abstract

Amyotrophic lateral sclerosis (ALS) is characterized by adult-onset progressive degeneration of upper and lower motor neurons. Increasing numbers of genes are found to be associated with ALS; among those, the first identified gene, *SOD1* coding a Cu/Zn-superoxide dismutase protein (SOD1), has been regarded as the gold standard in the research on a pathomechanism of ALS. Abnormal accumulation of misfolded SOD1 in affected spinal motor neurons has been established as a pathological hallmark of ALS caused by mutations in *SOD1* (*SOD1*-ALS). Nonetheless, involvement of wild-type SOD1 remains quite controversial in the pathology of ALS with no *SOD1* mutations (non-*SOD1* ALS), which occupies more than 90% of total ALS cases. In vitro studies have revealed post-translationally controlled misfolding and aggregation of wild-type as well as of mutant SOD1 proteins; therefore, SOD1 proteins could be a therapeutic target not only in *SOD1*-ALS but also in more prevailing cases, non-*SOD1* ALS. In order to search for evidence on misfolding and aggregation of wild-type SOD1 in vivo, we reviewed pathological studies using mouse models and patients and then summarized arguments for and against possible involvement of wild-type SOD1 in non-*SOD1* ALS as well as in *SOD1*-ALS.

## Background

Amyotrophic lateral sclerosis (ALS) is an adult-onset neurodegenerative disease classically characterized by loss of motor neurons in the central nervous system including motor cortex, brainstem, and spinal cord [1]. The loss of motor neurons leads to inability to control voluntary muscles and ultimately results in respiratory failure. Only two drugs, Riluzole and Edaravone, are currently available, but their therapeutic effects are limited to the extent that the survival can be extended at most a few months [2]. Together with full elucidation of the pathomechanism, therefore, development of efficient cures for this devastating disease has long been demanded.

In 1993, mutations in the gene encoding Cu/Zn-superoxide dismutase (SOD1) were first reported as a cause of ALS [3], and since then, more than 30 genes responsible for ALS have been identified [1]. A genetic cause/predisposition still remains unclear in most of ALS cases (~ 80%), and *SOD1* mutations describe only approximately 3% of total ALS cases (called *SOD1*-ALS) [4]. Nonetheless, pathological examinations on *SOD1*-ALS cases provide us with important clues to understand disease mechanisms; namely, SOD1 proteins abnormally accumulate and form inclusions selectively in affected motor neurons [5]. Based upon such pathological observations, furthermore, a mechanism has been proposed where SOD1 proteins assume an abnormal conformation (or misfold) by an amino acid substitution corresponding to a pathogenic mutation, accumulate as oligomers/aggregates, and then exert toxicity to kill motor neurons [6]. Several researchers have attempted to extend the pathological roles of SOD1 misfolding in *SOD1*-ALS to more prevailing ALS cases, in which no mutations in the *SOD1* gene are confirmed (non-*SOD1* ALS). In other words, wild-type SOD1 could cause ALS when it somehow misfolds. Nonetheless, experimental results on the involvement of wild-type SOD1 in non-*SOD1* ALS are not consistent among different research groups, making this issue highly controversial. In order to discuss SOD1 proteins as a potential target for the development of therapeutics to ALS, we comprehensively reviewed reports on possible roles of wild-type SOD1 in the pathology of ALS.

### Misfolded forms of SOD1 as a pathological hallmark of *SOD1*-ALS

SOD1 is a metalloenzyme that catalyzes the disproportionation of superoxide anion into hydrogen peroxide and molecular oxygen [7]. The enzymatic activity in most of the patients with the *SOD1* mutations was almost half as much as those in healthy controls [8], which had initially been considered to trigger pathological changes in ALS. Indeed, homozygous and even heterozygous knockout of the *Sod1* gene in mice exhibited a wide range of phenotypes relevant to ALS such as slowly progressive motor deficits [8]. Recently, furthermore, human patients with a homozygous truncating variant c.335dupG (p.C112Wfs*11) in the *SOD1* gene that leads to total absence of the enzymatic activity were reported, and the resulting phenotype was marked by progressive loss of motor abilities [9, 10]. Heterozygous carriers of the c.335dupG variant had an approximately halved SOD1 activity when compared to normal controls but appear not to develop symptoms of ALS [10]. Also, the *Sod1*-knockout mice did not develop ALS-like pathologies [8]; instead, overexpression of mutant SOD1 in mice reproduces ALS-like pathological changes with a significant increase in the SOD1 enzymatic activity [11]. While any reduction in the SOD1 enzymatic activity might modify the ALS pathomechanism, mutant SOD1 is considered to cause the disease not through a loss of the enzymatic activity but by a gain of new properties exerting toxicity to motor neurons.

As a pathological hallmark of *SOD1*-ALS, SOD1 proteins are known to abnormally accumulate in motor neurons (e.g. [5]), leading to prevailing idea that pathogenic mutant SOD1 gains toxicity through its misfolding into non-native conformations. While the abnormal accumulation of SOD1 in motor neurons does not necessarily mean the misfolding of SOD1, biophysical examinations in vitro using recombinant SOD1 proteins have strongly supported conformational changes of SOD1 by amino acid substitutions due to the pathogenic mutations. SOD1 is functionally and conformationally matured through post-translational processes including copper and zinc binding and disulfide formation [12]. The bound copper ion acts as a catalytic center, whereas the bound zinc ion and the intramolecular disulfide bond play roles in stabilizing the native structure [13–15]. Pathogenic mutations decrease the affinity of SOD1 toward the metal ions and/or the stability of the disulfide bond [16, 17], thereby disturbing the native conformation of SOD1. In other words, the post-translational maturation appears to be hampered in the mutant SOD1 proteins, resulting in an increased propensity of SOD1 to misfold into oligomers and aggregates. Indeed, in transgenic mice expressing human SOD1 with ALS-causing mutations (G37R and G93A), oral administration of a copper complex Cu^II^ (atsm) facilitates the copper binding of mutant SOD1 in their spinal cords and improves the neurological phenotype and survival [18–20]. Also, further expression of CCS, which is a copper chaperone assisting the maturation of SOD1 in vivo [21, 22], remarkably extends the survival of the transgenic mice administered with Cu^II^ (atsm) [23]. In the absence of the Cu^II^ (atsm) administration, overexpression of CCS in the transgenic mice (G37R and G93A) is known to dramatically reduce the mean survival (from 242 days to 36 days), to which mitochondrial dysfunction appears to contribute due to the perturbation of intracellular copper dynamics [24, 25]. Increased amounts of CCS would supply most of the intracellular copper ions to overexpressed mutant SOD1 proteins; therefore, the copper ions are not recruited to the other copper-requiring enzymes such as cytochrome *c* oxidase in mitochondria. Indeed, overexpression of CCS did not influence the disease phenotypes of the transgenic mice expressing human SOD1 with L126Z or murine SOD1 with G86R mutation [24], which are considered to be unable to bind a copper ion. Also notably, marked acceleration of disease in the transgenic mice (G93A) with CCS overexpression was not observed when the mice had an additional mutation H80G in the *SOD1* (G93A) transgene [26]. This is probably because the zinc-binding in G93A-mutant SOD1 was compromised by substitution of a zinc-ligand (His80) to Gly. Given important roles of the zinc binding in conformational stabilization of SOD1 [14, 27], H80G/G93A-mutant SOD1 was not able to receive a copper ion from the overexpressed CCS. Misfolding of SOD1 proteins in vivo as well as in vitro will hence be circumvented through their post-translational maturation of SOD1, which would eventually reduce the toxicity of mutant SOD1 proteins.

### Pathological roles of wild-type human SOD1 in transgenic mouse models of *SOD1*-ALS

Given that wild-type SOD1 is misfolded in vitro when losing the bound metal ions and/or the conserved disulfide bond [28], SOD1 could exert the disease-causing toxicity even without the pathogenic amino acid substitutions. Actually, co-expression of wild-type human SOD1 in transgenic mice expressing ALS-linked mutant human SOD1 (G37R, G85R, G93A, and L126Z) is known to accelerate the disease onset, suggesting the toxicity of wild-type human SOD1 [29–35]. Also, mice did not develop ALS-like symptoms upon expression of A4V-mutant human SOD1, but co-expression of wild-type human SOD1 in the A4V-SOD1 expressing mice did trigger the progression of ALS-like disease [29]. Taking advantage of distinct electrophoretic mobilities of wild-type and mutant SOD1 proteins (G85R and L126Z), furthermore, wild-type human SOD1 was found to accumulate as detergent-insoluble aggregates with the mutant proteins in transgenic mice [29, 31, 33, 34], while the interactions in the aggregates would not be simply a co-assembly of mutant and wild-type proteins [33]. A mechanism of disease-accelerating effects of wild-type SOD1 remains unclear, but heteromeric interactions between wild-type and mutant SOD1 appear to aggravate the aggregation and toxicity in cultured cell models [36] and have correlation with the disease severity [37]. It should be also noted that, in some studies, overexpression of wild-type human SOD1 did not affect the onset or duration of disease in mice expressing G85R-mutant human SOD1 [5] or G86R-mutant murine SOD1 [38]. Furthermore, disease-related phenotypes were not observed in transgenic mice expressing human SOD1 that has multiple mutations including those at copper and zinc binding sites (H46R/H48Q/H63G/H71R/H80R/H120G) and two free Cys residues (C6G/C111S) with an ALS-linked mutation, H43R, and co-expression of wild-type human SOD1 did not cause the disease [35]. Such apparent discrepancies would, nonetheless, indicate that expression levels of SOD1 as well as interactions between wild-type and mutant SOD1 play key roles in exerting toxicity of wild-type human SOD1.

Even in the absence of ALS-causing mutant SOD1, overexpression of wild-type human SOD1 alone can exert motor neuron toxicity to mice. In hemizygous transgenic mice expressing wild-type human SOD1, their lifespan was not affected, but neurodegenerative changes appeared in old age including mitochondrial vacuolization, axonal degeneration and a moderate loss of spinal motor neurons [32, 39, 40]. Upon decreasing glutathione levels, the mice developed overt motor symptoms, and their lifespan was decreased [41]. Also, spinal cord homogenates from the hemizygous wild-type human SOD1 transgenic mice were found to contain age-dependent, progressive formation of high-molecular-weight SOD1 aggregates [40, 42], which would be caused by oxidation of a unique tryptophan in SOD1 upon endoplasmic reticulum stress [42]. Furthermore, homozygous wild-type human SOD1 transgenic mice significantly increased the expression levels of wild-type human SOD1 and thereby developed ALS-like syndrome with formation of aggregated SOD1 in spinal cord and brain [43]. Even without any amino acid substitutions, therefore, wild-type human SOD1 could exert motor neuron toxicity to model animals under certain experimental conditions.

### Possible involvement of wild-type SOD1 in pathological inclusions of *SOD1*-ALS patients

In contrast to the mouse models, pathological involvement of wild-type SOD1 is highly controversial in *SOD1*-ALS as well as non-*SOD1* ALS patients. While most of *SOD1*-ALS patients express both wild-type and mutant SOD1 proteins, it is difficult to biochemically and immunohistochemically distinguish between wild-type and mutant SOD1 in tissues. In that sense, the involvement of wild-type SOD1 was examined in a *SOD1*-ALS patient with the G127insTGGG (G127X) mutation; such a truncated G127X-mutant SOD1 can be discriminated from the wild-type protein because of the difference in size and also of a non-native procession of the five amino acids following Gly127 in the variant [44, 45]. Wild-type SOD1 was detected in a detergent-insoluble (0.1% Nonidet P-40-insoluble) fraction of the cervical ventral horn of the G127X patient, while no control patients were examined [45] . Also, G127X patients had aggregates in glial cell nuclei of spinal cords, some of which were stained with an antibody (Chi 131–153 ab) raised against a peptide sequence absent in G127X-mutant SOD1 (Asn131 - Gln153) [46]. Those Chi 131–153 ab-positive aggregates were not stained with a G127X-mutant specific antibody directed to the non-native, C-terminal sequence of the five amino acids, suggesting pathological aggregation of wild-type SOD1 that is not co-localized with G127X-mutant proteins. As discussed later, however, even in control patients, significant amounts of wild-type SOD1 were present in the 0.1% Nonidet P-40-insoluble fraction [47]. Also, the same research group has published the paper showing that G127X-mutant but not wild-type SOD1 in the ventral horn of lumbar spinal cord of a G127X patient was sedimented by density gradient ultracentrifugation [44], implying no involvement of the wild-type protein in the mutant SOD1 aggregates. Some of the pathogenic full-length as well as truncated mutant SOD1 proteins are known to exhibit distinct electrophoretic mobilities from that of the wild-type protein [48]; therefore, more biochemical analysis on tissue samples from *SOD1*-ALS patients will reveal any involvement of wild-type SOD1 in the abnormal accumulation of SOD1 proteins in spinal cord.

### Controversies on pathological involvement of wild-type SOD1 in non-*SOD1* ALS

Also in non-*SOD1* ALS cases, which are much more prevailing than *SOD1*-ALS, there are harsh controversies on pathological roles of wild-type SOD1. While few studies have examined the metal binding and/or disulfide status of wild-type SOD1 in ALS, the lack of such post-translational processes is expected to result in the decrease of its enzymatic activity. Indeed, SOD1 activity in brain homogenates of sporadic ALS cases was reported to be decreased [49], but another study confirmed little differences in the activity in several parts of the central nervous system between sporadic ALS cases and non-ALS controls [50]. It should be noted that only the activity but not the amount of SOD1 was compared in those previous reports; therefore, it remains to be concluded whether wild-type SOD1 becomes misfolded and enzymatically inactive under pathological conditions of ALS.

SOD1 is ubiquitously and highly (10–100 μM) expressed as a soluble protein [51–53] (Human Protein Atlas available from http://www.proteinatlas.org) and diffusedly detected in most of subcellular compartments including cytoplasm [54], mitochondria [55], nucleus [56], and endoplasmic reticulum [57]. Based upon many studies using mouse models as well as purified proteins (e.g. [14, 58]), a consensus has been reached on the significantly reduced solubility of SOD1 by ALS-causing mutations, which leads to the formation of detergent-insoluble SOD1 aggregates. It should, however, be noted that only a few studies confirmed the solubility changes of SOD1 proteins in spinal cord tissues of ALS patients (even in those of *SOD1*-ALS patients).

**Fig. 1:**
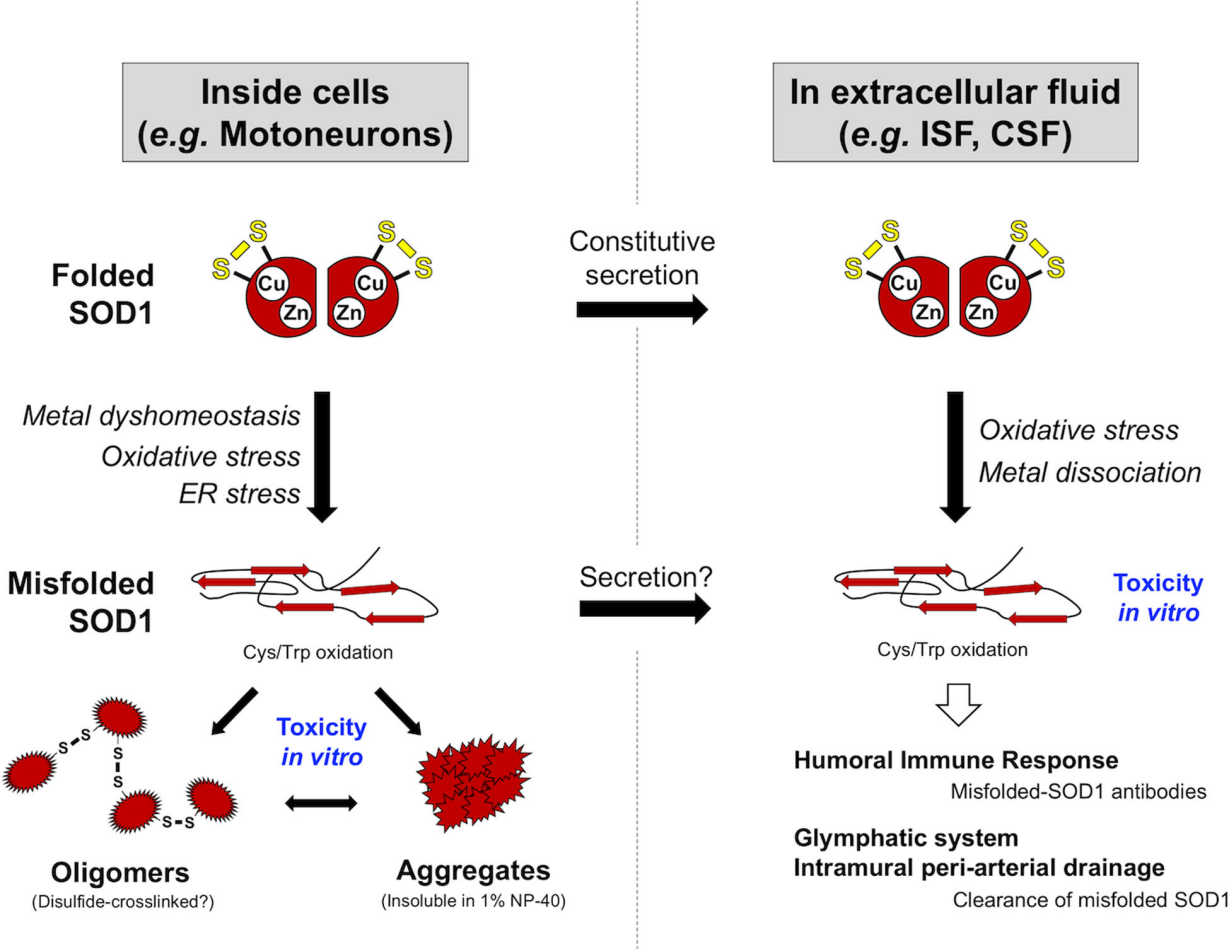
Schematic representation on possible changes of wild-type SOD1 in ALS. (Left) A natively folded SOD1 binds copper and zinc ions and forms an intramolecular disulfide bond. Pathological conditions might disrupt intracellular metal homeostasis and augment oxidative stress/ER stress, facilitating the formation of misfolded SOD1 even without any disease-causing mutations. Disulfide-crosslinked oligomers and insoluble aggregates of wild-type SOD1 have been detected in spinal cords of sporadic ALS. (Right) SOD1 has been known to constitutively secreted to extracellular fluid such as ISF and CSF, and recently, toxic wild-type SOD1 in abnormally misfolded conformations was detected in CSF of sporadic ALS. Misfolded SOD1 appears to be cleared by humoral immune response and/or glymphatic/intramural peri-arterial drainage systems, and their failure might contribute to the disease.

Bosco et al. prepared insoluble pellets from spinal cord homogenates in detergent-free lysis buffer, where comparable levels of SOD1 proteins were detected among a *SOD1*-ALS case (A4V mutation), four sporadic ALS cases, and four non-neurological controls [59]. No differences were observed in the amount of 0.1% Nonidet P-40-resistant SOD1 among two *SOD1*-ALS patients with the homozygous D90A mutations and two controls [47]. In contrast, when spinal cord homogenates were treated with 0.5% Nonidet P-40, significantly more amounts of SOD1 were detected in the insoluble fraction of a *SOD1*-ALS case (A4V mutation) than those of two familial ALS cases with unknown genetic causes, 12 sporadic ALS cases, and three controls [60]. Significantly more amounts of SOD1 were also detected in the 1% Nonidet P-40-insoluble pellets from two sporadic ALS cases (a non-*SOD1* ALS and a case with *C9orf72* mutation) as well as two *SOD1*-ALS cases (A4V and G72C mutations) than those of three Alzheimer’s disease cases and four non-neurological controls [61]. Furthermore, a filter-trap assay using a 0.22 μm cellulose acetate membrane was examined to detect SOD1 aggregates in spinal cord homogenates containing Nonidet P-40 and sodium dodecyl sulfate; wild-type SOD1 aggregates trapped on the membrane were significantly augmented in the lumbar spinal cord of sporadic ALS cases (4 positive/7 total) compared with control subjects (0 positive/6 total) [42]. It is thus possible that SOD1 proteins form detergent-insoluble aggregates in pathological conditions of ALS cases even without *SOD1* mutations (Fig. 1, left), but more numbers of studies will be required for conclusions.

Given that SOD1 is highly expressed in most of intracellular compartments, an immunohistochemical method using anti-SOD1 antibodies may be suitable for detection of pathological changes occurring in wild-type SOD1 only if the protein is densely accumulated as inclusion bodies. Indeed, a subset of Lewy body-like (hyaline) inclusions in the anterior horn cells of 10 out of 20 sporadic ALS patients (albeit with no test on *SOD1* mutations) were immunoreactive to anti-SOD1 antibodies, while skein-like inclusions and Bunina bodies were not [62–64]. Also, SOD1-immunoreactive inclusions were discerned against background staining in spinal cord motor neurons of a familial ALS patient without *SOD1* mutation [50]. In the other study, however, no SOD1-immunoreactivity was confirmed in the hyaline inclusions of all sporadic ALS cases examined (17 cases, again with no mention about *SOD1* mutations) [65]. While such a sharp discrepancy among those studies remains to be solved, different SOD1 antibodies were used for immunohistochemical analysis: a rabbit or sheep polyclonal antibody was raised against a holo form of human SOD1 in the former two studies [66], and a rabbit polyclonal antibody was raised against a SOD1 peptide corresponding to Asp124 to Lys136 in the latter [67]. These ideas are challenged by a report showing no SOD1-positive inclusions in non-*SOD1* ALS cases with a rabbit polyclonal anti-SOD1 antibody or a mouse monoclonal anti-SOD1 antibody [68]. Nonetheless, misfolding of SOD1 is well expected to affect epitope availability; therefore, the choice of the antibodies is still a key factor to detect any misfolded forms of SOD1 proteins in vivo. Indeed, increasing numbers of studies have examined non-*SOD1* ALS cases with conformation-specific antibodies that can discriminate misfolded SOD1 from the natively folded protein in vitro (called misfolded-SOD1 antibodies hereafter).

### Immunohistochemical examination on non-*SOD1* ALS cases with misfolded-SOD1 antibodies

As summarized in a recent comprehensive paper [69] as well as in an excellent review [70], a number of misfolded-SOD1 antibodies have been used for examination of sporadic ALS cases, and the results are sharply divided. In this review, we performed extensive search on the previous reports describing immunohistochemical and/or immunofluorescence examinations on human spinal cord tissues with misfolded-SOD1 antibodies, which is summarized in Table 1. As colored cyan in Table 1, some studies have claimed positive immunostaining of spinal cords (motor neurons and glial cells) selectively in sporadic and familial ALS with misfolded-SOD1 antibodies [46, 50, 59, 61, 69, 73–75]. As reviewed later in detail, a misfolded-SOD1 antibody (α-miSOD1) designed based on an antibody from the healthy elderly subjects was also found to stain spinal cord of sporadic as well as familial ALS patients but not of non-neurological controls [71]. In the other studies (colored orange in Table 1), however, no difference in the staining pattern was observed between ALS and non-ALS controls [72, 74, 76–79]. Some of the misfolded-SOD1 antibodies in Table 1 (in particular, the ones reported from one research group: SEDI, USOD, AJ10, B8H10, 4A1, and A5E5) were found to immunostain spinal motorneurons in *SOD1*-ALS but not in non-*SOD1* ALS, which might simply mean that misfolded conformations of wild-type SOD1 in non-*SOD1* ALS are not the same with those of mutant SOD1 in *SOD1*-ALS. Immunostaining results using mouse monoclonal C4F6, 3H1, 10E11C11 and a rabbit polyclonal Ra 131–153 antibody have been reported from more than two research groups but still did not reach a consensus about the detection of misfolded SOD1 in non-*SOD1* ALS cases (Table 1). Much effort has been directed to resolve those discrepancies, which could be caused by differences in experimental procedures including tissue fixation, antigen retrieval, and working concentrations of primary antibodies [69]. Indeed, antigen retrieval treatments in a citrate buffer with heat (boiling, steaming, microwave) are considered to denature SOD1 proteins, which could efficiently expose the epitope for misfolded-SOD1 antibodies [72] but appears not to describe the discrepancy on the immunohistochemical detection of misfolded SOD1 (Table 1).

**Table 1:**
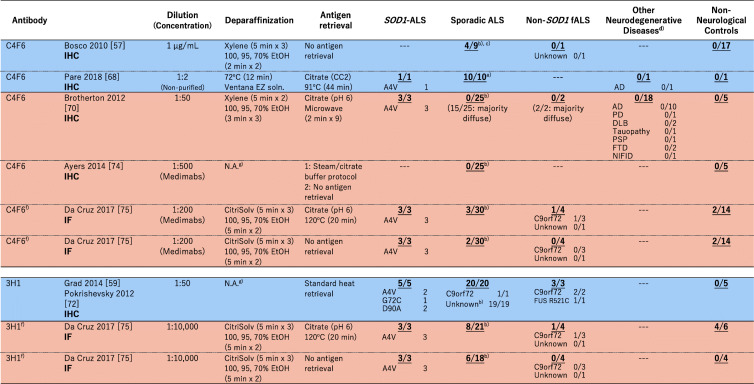

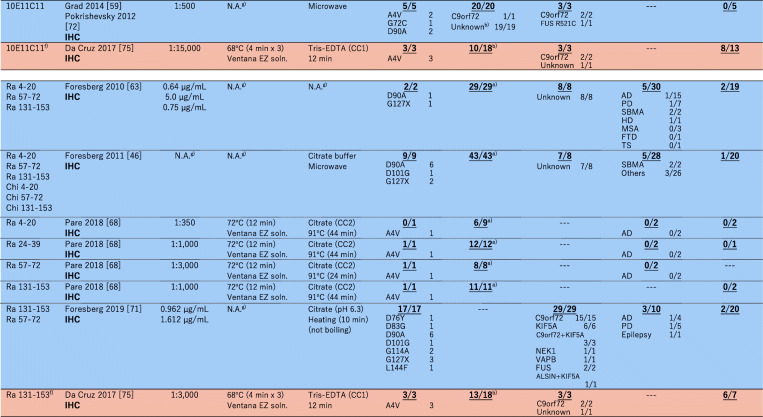

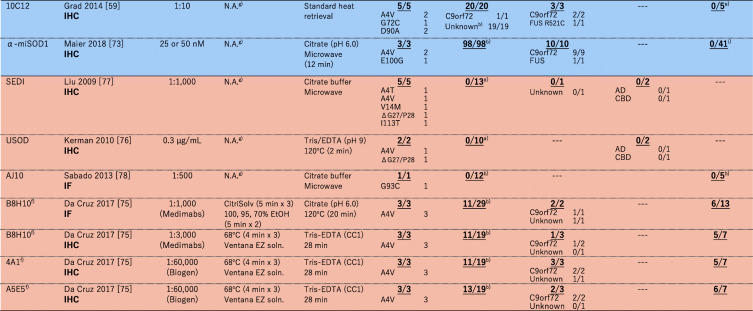
A summary on immunohistochemical (IHC)/immunofluorescence (IF) detection of misfolded SOD1 with misfolded-SOD1 antibodies in human spinal cords. The numbers in each box represent (the number of misfolded-SOD1-positive cases)/(the number of total cases examined)

^a^No *SOD1* mutations were confirmed. ^b^There is no mention on the presence or absence of *SOD1* mutations. ^c^No motor neurons in the five remaining cases. ^d^*AD*, Alzheimer’s disease, *PD* Parkinson’s disease, *DLB* Dementia with Lewy body, *PSP* Progressive supranuclear palsy, *FTD* Frontotemporal dementia, *NIFID* neuronal intermediate filament inclusion disease, *SBMA* Spinal and bulbar muscular atrophy, *HD* Huntington’s disease, *MSA* Multiple system atrophy, *TS* Tuberous sclerosis, *CBD* Corticobasal degeneration. ^e^There is no mention on the non-neurological controls in the paper. ^f^In this review, the cases with cytoplasmic granular staining, rare round deposits, abundant round deposits, and globular inclusions are counted as misfolded-SOD1 positive, while the cases with no signal, sparse diffuse staining, and abundant diffuse staining are counted as misfoded-SOD1 negative. ^g^Not available (no mention in the paper). ^h^The control cases are described as “non-ALS controls”. ^i^In the paper, it was described that “no or only weak immunoreactivity was observed in motor neurons of most of the 41 spinal cord tissue samples from NNC patients”

In immunohistochemical/immunofluorescence analysis of tissues, the experimental procedures/conditions are often not described in detail; in particular, a working concentration of a primary antibody is usually indicated as a dilution factor but not a concentration of the antibody in many studies. These situations prevent us from comparing the previously reported staining results in detail; based upon Table 1, however, a trend can be found that a significant dilution of the misfolded-SOD1 antibodies fails to detect non-*SOD1* ALS-specific immunostaining. The antibody C4F6 is commercially available from MediMabs, and the concentration was found to be < 0.05 mg/mL in our hands. Ayers et al. [72] and Da Cruz et al. [77] have reported the absence of C4F6-positive staining in sporadic ALS cases by using the C4F6 antibody from MediMabs in 500-fold and 200-fold dilution (Table 1), which would correspond to < 0.1 and < 0.25 μg/mL of the working concentration, respectively. Instead, Bosco et al. successfully detected C4F6-positive staining with 1.0 μg/mL C4F6 in some of sporadic ALS cases but not in non-neurological controls (Table 1) [59]. Also in the three papers by Grad et al. [61], Pokrishevsky et al. [75], and Da Cruz et al. [77], we have supposed that they used the antibodies 3H1 and 10E11C11 originated from the same source for immunohistochemical examination on misfolded SOD1 (we further assumed the same concentration of the original antibody solution in their studies). Successful detection of misfolded SOD1 in ALS tissues with a lower dilution rate of the antibodies was reported by Grad et al. and Pokrishevsky et al., but Da Cruz et al. appear to have used a significantly diluted solution of the antibody and failed to detect the non-*SOD1* ALS-specific, 3H1- and 10E11C11-positive staining. Furthermore, Brännström group prepared a polyclonal antibody Ra 131–153 for detection of misfolded SOD1 proteins and observed the Ra 131–153-positive immunostaining in non-*SOD1* ALS as well as *SOD1*-ALS cases [46, 50, 69, 73]. The antibody was then distributed to the other research group and used for the immunohistochemical examination; however, the Ra 131–153-positive immunostaining was observed in not only ALS but also non-neurological control cases [77], which might be due to an antigen retrieval step using Tris-EDTA-based solution [69]. Collectively, further investigations with more quantitative, detailed descriptions on the experimental procedures (a working concentration of antibodies, in particular) will be definitely required for evaluating immunohistochemical evidence of misfolded SOD1 proteins in non-*SOD1* ALS cases.

### Immunoprecipitation from spinal cords of non-*SOD1* ALS with misfolded-SOD1 antibodies

Immunohistochemical examinations require several harsh treatments of tissue samples (depaffinization, antigen retrieval, etc.) that can significantly affect protein conformations; therefore, the presence or absence of misfolded wild-type SOD1 proteins in tissues may not be accurately evaluated. Instead, more accurate evidence on misfolded wild-type SOD1 in ALS could be provided by immunoprecipitation (IP) from unfixed spinal cord homogenates with misfolded-SOD1 antibodies, which are summarized in Table 2. Again, experimental details required for testing reproducibility were not fully described in most of the papers, and the results were sharply divided. Mutant SOD1 in all *SOD1*-ALS cases examined was successfully immunoprecipitated with any of misfolded-SOD1 antibodies listed in Table 2, and wild-type SOD1 in sporadic ALS cases without *SOD1* mutations was also immunoprecipitated in the studies by Grad et al. [61] and Paré et al. [69]. In contrast, the other studies by Liu et al. [78], Kerman et al. [76], and Da Cruz et al. [77] have concluded that no wild-type SOD1 proteins are immunoprecipitated from spinal cords of sporadic ALS cases with misfolded-SOD1 antibodies. Nonetheless, we note that the interpretation on the immunoprecipitation results appears somewhat different among those studies; namely, no SOD1 proteins were observed in immunoprecipitates from sporadic ALS with SEDI (Liu et al. [78]) and USOD (Kerman et al. [76]) antibodies, while the misfolded-SOD1 antibodies (3H1, 4A1, A5E5) used in the Da Cruz et al. paper did immunoprecipitate SOD1 proteins in sporadic ALS cases but also in non-neurological controls [77]. Using the 3H1 antibody, furthermore, Grad et al. were found to immunoprecipitate wild-type SOD1 from spinal cords of sporadic ALS cases but not from those of non-neurological controls [61]. Again, it is highly possible that some differences in experimental procedures influence the detection of misfolded wild-type SOD1 in sporadic ALS tissues, and much more numbers of studies with detailed description on IP methods are definitely required.

**Table 2:**
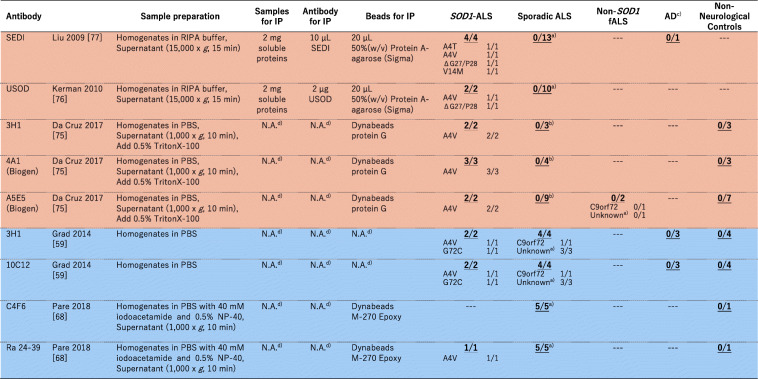
A summary on immunoprecipitation of misfolded SOD1 with misfolded-SOD1 antibodies from human spinal cords. The numbers in each box represent (the number of misfolded-SOD1-positive cases)/(the number of total cases examined)

^a^No *SOD1* mutations were confirmed. ^b^There is no mention on the presence or absence of *SOD1* mutations. ^c^Alzheimer’s disease. ^d^Not available (no mention in the paper)

It is also important to note that wild-type SOD1 immunopurified with anti-SOD1 antibody from spinal cord homogenates of sporadic ALS inhibited anterograde but not retrograde fast axonal transport in the assay using isolated squid axoplasm through a mechanism possibly involving specific activation of p38 MAPK [59]. Such inhibition was no longer observed when the immunopurified SOD1 proteins were first mixed with the misfolded-SOD1 antibody C4F6 and then perfused into squid axoplasm. These results have thus supported toxic and pathogenic roles of misfolded wild-type SOD1 in sporadic ALS (Fig. 1, left).

### Misfolded forms of SOD1 in cerebrospinal fluid of ALS

As described, SOD1 is localized mostly in the cytoplasm (Human Protein Atlas, see above), and the intraneuronal inclusions containing SOD1 are the pathological hallmark of *SOD1*-ALS [5]. Many researchers thus focused on the toxic/conformational properties of SOD1 within cells, even though SOD1 proteins were reported to be present also in the extracellular space by their active and constitutive secretion from cells (Fig. 1, upper) [80, 81]. Recently, misfolded/aggregated proteins are considered to propagate between cells, which would contribute to the pathological progression in many of neurodegenerative diseases including *SOD1*-ALS [82–86]. For example, premature motor neuron disease in transgenic mice expressing human SOD1 with G85R mutation is triggered by inoculation of detergent-resistant fractions of SOD1 from a *SOD1*-ALS patient (G127Gfs*7) into the lumbar spinal cord [83]. Also, much attention has been paid on glymphatic system [87] and intramural peri-arterial drainage pathway [88], by which misfolded/aggregated proteins in interstitial fluid (ISF) of the brain and spinal cord could be drained into cerebrospinal fluid (CSF) and then cleared [89]. Regarding *SOD1*-ALS, indeed, the disease duration of transgenic mice expressing ALS-linked mutant SOD1 was shortened by deletion of aquaporin-4 [90], a water channel playing central roles in the extracellular clearance through the glymphatic system [87]. Furthermore, pathologies and amyloid-β accumulation in transgenic mouse models of Alzheimer’s disease were aggravated by disrupting meningeal lymphatic vessels, which are proposed as a drain of macromolecules from ISF and CSF [91]. Therefore, SOD1 proteins that are secreted from neurons and glia and then possibly drained into CSF will be important in understanding the pathology of ALS.

Indeed, SOD1 is well known as a constituent of CSF, and amounts of SOD1 in CSF tended to increase as a function of age albeit with a low correlation coefficient (*r*^2^ = 0.1 ~ 0.2) [92–94]. In most studies, total SOD1 levels in CSF appear to be not significantly different between ALS and neurological/non-neurological controls [92–96]. Alternatively, absolute levels of SOD1 in CSF were reported to show substantial variability among individuals but with little variability in each individual over time [97]. In the same study [97], ALS cases and neurological controls were characterized by slightly higher levels of SOD1 in CSF compared to those of healthy controls; however, the amount of SOD1 in CSF did not correlate with the severity of ALS. In CSF, significant fractions of SOD1 were also reported to be N-terminally truncated, but the amount of such truncated proteins did not differ between ALS and controls, suggesting little pathological roles of the truncated SOD1 in ALS [93, 95]. In electrophoretic analysis of CSF, furthermore, neither SOD1-positive smears nor high-molecular-weight ladders were observed, indicating that detergent-resistant oligomers/aggregates were not evident in CSF of ALS [93, 95]. Based upon those reports, SOD1 in CSF appears to have no pathological roles in ALS. Nonetheless, it is quite notable that, in rats overexpressing wild-type human SOD1, half-life of the SOD1 protein was significantly longer in CSF (14.9 days) as well as in spinal cord (15.9 days) than that in liver and kidney (1.7 and 3.4 days, respectively) [98]. Also in CSF of human subjects, the turnover rate of SOD1 was found to be significantly slower (half-life: 25.0 +/− 7.4 days) than that of total proteins (half-life: 3.6 +/− 1.0 days) [98]. Accordingly, slow turnover rate of SOD1 in CSF as well as in spinal cord would allow sufficient time for SOD1 to become misfolded and to contribute to the development of pathological changes.

To test if SOD1 becomes misfolded in CSF of ALS, CSF samples from 96 ALS cases (57 sporadic ALS, 22 *SOD1*-ALS, 17 Non-*SOD1* familial ALS) and 38 neurological controls were examined with sandwich ELISA using misfolded-SOD1 antibodies (Ra 24–39, Ra 57–72, and Ra 111–127) [94]. Signals indicating the presence of misfolded SOD1 were found in all samples, but no significant differences were confirmed between ALS with and without *SOD1* mutations and also between the ALS cases combined and the controls [94]. In contrast, by using other types of misfolded-SOD1 antibodies, we recently showed that wild-type SOD1 proteins were misfolded in CSF of sporadic ALS cases as well as of a *SOD1*-ALS case [95]. More precisely, sandwich ELISA was performed on CSF from 21 ALS cases (20 sporadic ALS, 1 *SOD1*-ALS) and 40 controls by using misfolded-SOD1 antibodies (C4F6, UβB, EDI, apoSOD, 24–39 and SOD1^int^). Among those, C4F6, UβB, EDI, and apoSOD were found to give significantly higher signals in CSF of ALS cases compared to those of controls; in contrast, no differences were observed with 24–39 and SOD1^int^. It was also surprising to us that large fractions of SOD1 in CSF of sporadic ALS cases were immunoprecipitated with C4F6 antibody [95]. CSF collected from ALS patients has been known to exert toxicity toward motor-neuron like cells NSC-34 [99], and we revealed that the toxicity was alleviated by removing the misfolded SOD1 from CSF with immunoprecipitation using C4F6 antibody [95]. It is also notable that misfolded SOD1 immunoreactive to C4F6 and UβB was observed, albeit with less amount, in CSF of a subset of patients with Parkinson’s disease (PD) and progressive supranuclear palsy (PSP). Therefore, not all types of misfolded-SOD1 antibodies could detect pathological forms of wild-type SOD1 in CSF, but our study has suggested that wild-type SOD1 in CSF adopts a misfolded, toxic conformation(s) in pathological conditions of ALS and also a subset of PD and PSP. In that sense, it is important to note that levels of SOD1 in CSF of *SOD1*-ALS patients were reduced by oral medication with pyrimethamine [100].

### Misfolding of wild-type SOD1 under oxidative environment of spinal cord and CSF

Another important issue to be solved is where SOD1 is misfolded; in other words, it remains to be tested whether SOD1 is misfolded in CSF, or misfolded SOD1 in affected spinal cord (or some other tissues) is drained into CSF (Fig. 1). As of now, we do not have an answer to this question; nonetheless, one of the notable features observed commonly in spinal cord and CSF of ALS patients is significantly elevated levels of oxidative markers, which has been summarized in an excellent review [101]. It is thus plausible that oxidative environment in the spinal cord/CSF of ALS is important to understand any pathological changes occurring in SOD1.

In accordance with this, we have detected abnormal SOD1 oligomers crosslinked via intermolecular disulfide bonds in spinal cord of *SOD1*-ALS cases as well as transgenic mice expressing human SOD1 with ALS mutations (G37R, G93A, and L126Z) [31, 102]. While the disulfide-crosslinked SOD1 oligomers were not evident in CSF of sporadic ALS cases and a *SOD1*-ALS case [95], reductant (DTT)-sensitive aggregates of wild-type SOD1 were detected in affected spinal cord of sporadic ALS cases [42]. Furthermore, Xu et al. suggested the oxidation of Cys111 in SOD1 to a sulfenic acid (−SOH) in CSF of a subset of sporadic ALS cases [103], and we also found that Cys111 was oxidized to a sulfonic acid (−SO_3_H) in CSF of a subset of ALS, PD, PSP, and AD cases [95]. In our experiments in vitro [104], followed by the sulfenylation of Cys111 in metal-bound SOD1 with H_2_O_2_, dissociation of the bound metal ions from the protein was found to allow another free Cys residue (Cys6) to attack the sulfenylated Cys111. SOD1 has a canonical intramolecular disulfide bond between Cys57 and Cys146; therefore, oxidation with H_2_O_2_ led to the formation of abnormal SOD1 (SOD1^2xS-S^) with two intramolecular disulfide bonds (Cys6-Cys111 and Cys57-Cys146), and SOD1^2xS-S^ was prone to aggregation and also toxic to motor-neuron like cells NSC-34 [104].

As summarized above, Cys is considered to be the most susceptible to oxidation among amino acids and would hence be a key residue for oxidative modifications under pathological conditions. Notably, several other oxidized forms of SOD1 have been also reported in cell lines, transgenic mice, and purified SOD1 proteins. For example, SOD1 proteins with oxidized carbonyl groups were detected in lymphoblasts derived from sporadic ALS with bulbar onset [105]. SOD1 oxidized at tryptophan (Trp32) was found to accumulate in the microsomal fractions purified from spinal cord of transgenic mice expressing wild-type human SOD1 [42] and was also detected in human blood and the blood isolated from transgenic mice expressing wild-type or ALS-linked mutant human SOD1 [106]. Furthermore, several His residues as well as Trp32 are also susceptible to oxidation, which has been proposed to trigger the aggregation of SOD1 in vitro [107–110]. It, however, remains to be tested whether the His and/or Trp oxidations occur on SOD1 in ALS patients.

### Misfolded SOD1 in extracellular fluid as a potential immunotherapeutic target

As reviewed above, formation of misfolded and plausibly toxic SOD1 species in extracellular fluid is well expected as a pathological change occurring in ALS cases. This could in turn open the way to alleviate the disease by removing such extracellular SOD1 proteins with the humoral immune response. Indeed, the survival of transgenic mice expressing ALS-linked mutant SOD1 was extended by vaccination with full-length misfolded SOD1 proteins [111, 112] and with peptides corresponding to the region available only in misfolded SOD1 [113, 114]. Passive immunization with several misfolded-SOD1 antibodies was also reported to be beneficial to the *SOD1*-ALS model mice [112, 115–117] except for one study [118]. Furthermore, sera from sporadic ALS patients were found to contain IgM antibodies reacting with misfolded SOD1 (recombinant SOD1 oxidized with 10 mM H_2_O_2_), and the sporadic ALS cases with higher levels of the IgM antibodies (*n* = 153) exhibited a longer survival of 6.4 years than the subjects lacking those antibodies (*n* = 127) [119].

Notably, Maier et al. screened human memory B cell repertoires from a large cohort of healthy elderly subjects and successfully generated a monoclonal antibody (α-miSOD1) that can react selectively with misfolded/oxidized SOD1 but not with native SOD1 [71]. Based upon the presence of B cell memory against misfolded SOD1 in a majority of those healthy elderly subjects, Maier et al. suggested that misfolding of SOD1 and the subsequent humoral immune response are frequent events in the elderly [71]. This antibody, α-miSOD1, was found to stain motor neurons of the spinal cord samples from ALS including sporadic as well as familial cases with and without *SOD1* mutations, but not from non-neurological controls (Table 1) [71]. Furthermore, intracerebroventricular infusion and also intraperitoneal injections of α-miSOD1 antibody to transgenic mice expressing ALS-linked mutant human SOD1 (G37R and G93A) delayed the onset of motor symptoms and extended survival [71]. Therefore, clearance of misfolded SOD1 by utilizing the immune system would be a potential treatment for patients with sporadic as well as familial ALS; nonetheless, it should be also noted that, in sera of sporadic ALS subjects, higher levels of IgG antibodies reacting with normal wild-type SOD1 associated with a shorter survival of 4.1 years [119]. For successful immunotherapy to treat ALS, it will be critical to develop antibodies specifically recognizing toxic, misfolded SOD1 and/or to design antigens efficiently producing such antibodies.

## Conclusions

While misfolding of ALS-linked mutant SOD1 has been established as a pathological change occurring in *SOD1*-ALS, roles of wild-type SOD1 in more prevailing non-*SOD1* ALS have long been debated. Even in *SOD1*-ALS, involvement of wild-type SOD1 in the pathology remains obscure. As reviewed above, we performed an extensive literature search and found that a number of studies supported the presence of misfolded wild-type SOD1 in spinal cord and CSF of non-*SOD1* ALS cases (Fig. 1). Nonetheless, not all studies detected misfolded wild-type SOD1 proteins in non-*SOD1* ALS, possibly suggesting the importance of experimental conditions in their immunohistochemical and immunochemical detection. Also, some of misfolded-SOD1 antibodies gave positive signals in *SOD1*-ALS but not in non-*SOD1* ALS, which may indicate distinct conformations of misfolded SOD1 between *SOD1*-ALS and non-*SOD1* ALS. As we recently reported [95], CSF of non-*SOD1* ALS contained misfolded forms of wild-type SOD1. The misfolded SOD1 in CSF was toxic to cultured cells, but it still needs to be tested whether it is a pathogenic species causing degeneration of motor neurons. Quite notably, misfolding of SOD1 could occur in the healthy elderly, and the humoral immune response to the misfolded SOD1 would be a key to prevent ALS. Consistent with beneficial results of immunization-based treatment of transgenic mouse models, therefore, immunological modulation of misfolded SOD1 in extracellular fluids such as CSF would be a promising strategy to delay onset and/or relieve symptoms of ALS.

## Data Availability

Not applicable.

## Abbreviations

ALS: Amyotrophic lateral sclerosis
CCS: Copper chaperone for SOD1
CSF: Cerebrospinal fluid
ISF: Interstitial fluid
non-*SOD1* ALS: Amyotrophic lateral sclerosis without mutations in the *SOD1* gene
PD: Parkinson’s disease
PSP: Progressive supranuclear palsy
SOD1: Cu/Zn-superoxide dismutase
*SOD1*-ALS: Amyotrophic lateral sclerosis with mutations in the *SOD1* gene
